# Influence of immunomagnetic enrichment on gene expression of tumor cells

**DOI:** 10.1186/1479-5876-3-12

**Published:** 2005-03-16

**Authors:** Ute Woelfle, Elisabeth Breit, Klaus Pantel

**Affiliations:** 1 Institute of Tumor Biology, University Medical Center Hamburg-Eppendorf, Hamburg, Germany

## Abstract

**Background:**

Metastasis is the leading cause of cancer-related death. Bone marrow (BM) is a frequent site for the settlement of disseminated tumor cells which occurs years before overt metastases signal incurability.

**Methods:**

Here we describe a new method to assess the initial stage of metastasis development in cancer patients. By immunomagnetic selection with HER2/neu and EpCAM as catcher antigens single disseminated tumor cells can be enriched from BM samples. To examine whether the immunomagnetic enrichment technique may change gene expression in the selected tumor cells, we performed differential expression profiling with the breast cancer cell lines MCF-7 and BT474 as models. The profiles were performed using 1.2 Cancer Arrays (Clontech) containing 1176 cDNAs that can be grouped into different functional categories, such as signal transduction, cell cycle, adhesion, cytoskeleton plasticity, growth factors and others.

**Results:**

The reproducibility of the gene expression profiling between duplicate cDNA-array experiments was assessed by two independent experiments with MCF-7 breast cancer cells. Scatter blot analysis revealed a good reproducibility of the cDNA array analysis (i.e. less than 10% difference in the gene expression between the arrays). Subsequent comparative cDNA-array analyses of immunobead-selected and unselected MCF-7 and BT474 cancer cells indicated that the antibody incubation during the immunomagnetic selection procedure did not considerably alter the gene expression profile.

**Conclusion:**

The described method offers an excellent tool for the enrichment of micrometastatic tumor cells in BM without largely changing the gene expression pattern of these cells.

## Introduction

Solid tumors derived from epithelial organs are the main form of cancer in industrialized countries. The first phase of the metastatic development consists of local tumor cell invasion, followed by tumor cell circulation in the blood and homing to secondary distant organs [1,2]. As indicator organ for early systemic dissemination of epithelial tumor cells to distant sites, BM has played a prominent role [3]. BM can easily be aspirated from the iliac crest and single metastatic cells are already present in 20–40% of patients with epithelial tumors (e.g., breast, lung or colon carcinomas) years before overt distant metastases occur in the skeleton or other distant organs [3-5]. The molecular description of these cells has been, however, hampered by the low concentration of these cells (e.g., 10^-5^-10^-6 ^per BM cell). To predict and monitor therapeutic responses the assessment of the gene expression profile of disseminated tumor cells seems to be of utmost importance. However, it is uncertain to which extent incubation with antibodies used for immunomagnetic isolation of these cells might affect their expression profile. We have addressed this aspect, using monoclonal antibodies against two prominent antigens, EpCAM and HER2/neu that are frequently and independently expressed on micrometastatic tumor cells [3].

## Materials and methods

### Ficoll density gradient centrifugation and immunocytochemistry

The enrichment of tumor cells from BM by Ficoll density gradient centrifugation and the immunocytochemical detection of epithelial tumor cells in cytological BM preparations has been described elsewhere in detail [5,6].

### Immunomagnetic cell separation and immunocytochemistry of BM samples

Two ml of a BM sample usually containing 2 × 10^6 ^mononuclear cells were washed with Hank's Salt Solution (Biochrom KG, Germany). The pellet was resuspended in 2 ml Hanks and 3.2 × 10^7 ^(80 μl) CELLection™ and pan-mouse immunomagnetic beads (Dynal, Oslo, Norway) coated with anti-EpCAM (MAb 3B10) and anti-HER2/neu (MAb 7C1) antibodies (Micromet, Munich, Germany) were added. All solutions and cell preparations were kept at 4°C during the whole procedure to avoid nonspecific binding of immunomagnetic beads. After an incubation time of 30 min at 4°C and 20 min at room temperature on a rotating mixer the magnetically labeled cells were isolated in a magnetic particle concentrator and resuspended in 200 μl bead removing buffer (40 mM Tris, 10 mM MgSO_4_, and 1 mM CaCl_2_, pH7.4, prewarmed to room temperature). The immunomagnetic beads were removed by DNase treatment with 15 μl DNase (50 U/μl) at room temperature for 15 min. After separation in a magnetic particle concentrator the supernatant was collected and centrifuged onto glass slides. Tumor cells were identified by immunostaining with monoclonal anti-cytokeratin antibody A45-B/B3 according to the manufacturer's instruction (Micromet, Munich, Germany). Cytokeratins are specific constituents of the epithelial cytoskeleton and they have become the marker antigen of choice for the detection of disseminated epithelial tumor cells in mesenchymal organs such as BM [3,7]. To avoid unspecific binding of the antibody via Fc-receptors present on leukocytes, we used F_ab _fragments of A45-B/B3 that were directly conjugated to the marker enzyme alkaline phosphatase.

### Cell culture and antibody incubation

MCF-7 cells and BT474 cells were maintained in RPMI (Invitrogen, Karlsruhe, Germany) supplemented with 5 % glutamine (Invitrogen) and 10 % FCS (C-C Pro, Neustadt, Germany). MCF-7 and BT474 cells (ATCC HTB-22 and HTB-20) were allowed to reach a logarithmic growth phase in culture. At 90 % confluency, the cells were incubated for 30 min at 4°C and 20 min at room temperature in Hanks containing 1 μg/ml of anti-EpCAM (MAb 3B10) as well as anti-HER2/neu (MAb 7C1) antibodies according to the immunomagnetic cell separation protocol. In a negative control experiment, cells were suspended in Hanks devoid of antibodies.

### cDNA probe preparation and hybridization

Total RNA was isolated using the peqGold TriFast™ (Peqlab, Erlangen, Germany) according to the manufacturer's instruction. In order to remove genomic DNA contamination, a DNase step was included using the DNA-free™ kit (Ambion, Cambrigeshire, England) according to manufacturer's instructions. RNA was dissolved in RNase-free H_2_O with 1 U/μl RNase inhibitor (SUPERase. IN™, Ambion). 5 μg purified total RNA was used for [α-^33^P] dATP (3000 Ci/mmol, 10 μl; Amersham, Freiburg, Germany) labeled cDNA synthesis as previously described [8]. The cDNA probe was purified with nucleotide removal columns (Qiagen, Hilden, Germany). The Atlas Human 1.2 Cancer Arrays (Clontech, Heidelberg, Germany) were hybridized according to the manufacturer's protocol.

### cDNA- array data analysis

The membranes were exposed to phosphoimager plates (Raytest Isotopen-Meβgeräte, Straubenhardt, Germany) for 3 days, and plates were scanned with the phosphoimager Fuji Bas (Raytest) at a 100 μm-resolution. The images were analyzed using the Imagene 5.5 software (Biodiscovery, CA, USA). The data of the arrays were normalized on the basis of the genes ubiquitin, HLAC and beta actin. The ratio between antibody-treated and non-treated cells was calculated for each gene. Ratios lower than 0.5 or higher than 2 were considered as differentially expressed if at least one sample showed an expression above 0.5. We performed duplicates of each experiment and created scatter blots with the SPSS software for windows.

## Results

**Figure 1:**
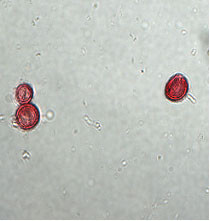
CK-positive cells detected after immunomagnetic enrichment of BM from breast cancer patients. One single cell and one 2-cell cluster is shown in a 400× magnification.

In a recent work [9] we demonstrated that our immunomagnetic separation works on clinical samples (Figure 1) and is superior to the standard Ficoll density centrifugation technique, used in most previous studies on cancer micrometastasis [4,5,7,10].

To test whether the antibody incubation during the immunomagnetic enrichment approach affects gene expression in the selected cells, we applied cDNA-array analysis. We subsequently evaluated whether the immunomagnetic enrichment method affected gene expression in the selected cells. This aspect is of utmost importance for further molecular description of disseminated tumor cells and has not been addressed before. The profiles were performed using 1.2 Cancer Arrays (Clontech) containing 1176 cDNAs that can be grouped into different functional categories, such as signal transduction, cell cycle, adhesion, cytoskeleton plasticity, growth factors and others [11].

**Figure 2:**
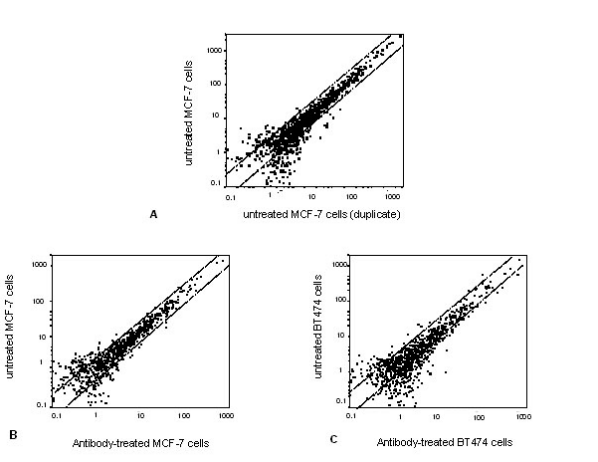
Representative scatter blots of breast cancer cells using the 1.2 Cancer Array for expression analysis. (A) untreated MCF-7 breast cancer cells (results of duplicate experiments), (B) antibody-treated versus untreated MCF-7 cells, and (C) antibody-treated versus untreated BT474 breast cancer cells.

As models, we used the breast cancer cell lines MCF-7 and BT474 (ATCC HTB-22 and HTB-20), because they express heterogeneous levels of the target antigens HER2/neu and EpCAM comparable to micrometastatic breast cancer cells in vivo [3]. The reproducibility of the gene expression profiling between duplicate cDNA-array experiments was assessed by two independent experiments with MCF-7 breast cancer cells. As shown in Figure 2A, scatter blot analysis revealed a good reproducibility of the cDNA array analysis (i.e. less than 10% difference in the gene expression between the arrays). We plotted the data of the antibody-treated and untreated MCF-7 (B) or BT474 (C) cells two dimensionally in a scatter plot; y-axis represents the data of untreated cells and the x-axis represents the data of cells treated with anti-HER2/neu or anti-EpCAM. For both cell lines, the scatter plots show that expressed genes in antibody-treated versus untreated cells (Figure 2B, C) was in principal within the range observed in the duplicate experiments with MCF-7 cells (Figure 2A). However, subtle changes in the expression of individual genes after antibody incubation were observed in particular in BT474 cells. In this cell line 38 genes were strongly differentially expressed (ratio >3) in antibody-treated versus untreated cells (Table 1). Most of these genes play a role in extracellular matrix remodeling, signal transduction and replication, as well as repair and transcription. MCF-7 cells showed in this experimental approach 31 differentially expressed genes with a ratio of over 3 (data not shown). Although similar group of genes were affected, only 3 common genes (CDC7, SGI and KIR) were differentially expressed in both cell lines after antibody incubation.

**Table 1:** Genes differentially expressed in antibody-treated versus untreated BT474 cell

**Genes**	**GenBank Accession#**	Ratio*
**Extracellular matrix remodeling**:		
*COL11*	J04177	- 6.2
*MMP17*	X89576	- 4.7
*MMP16*	D50477	-4.6
*SPARC*	J03040	5.7
		
**Adhesion:**		
*PKD1*	U24497	- 7.3
*NCAM*	AF002246	7.3
*M-cadherin*	D83542	3.5
		
**Cytoskeleton plasticity:**		
*SPTA1*	M61877	7.4
		
**Signal transduction:**		
*RGS4*	U27768	- 10.1
*GAS*	L13720	- 7.1
*BMP1*	U50330	- 7.0
*FGFR4*	L03840	- 6.5
*PMEL17*	M77348	- 6.5
*ETS-1*		- 5.9
*ERBB2*	M95667	- 5.8
*TGF-beta*	X02812	- 5.2
*N-ras*	X02751	- 4.8
*HRS*	D84064	- 4.7
*KIR*	U10550	9.0
*BMP6*	M60315	7.5
*BIN1*	U68485	7.4
*SGI*	Y00064	4.1
*CDC7*	AF015592	3.8
*CNTF*	S72921	3.4
*SH3BP2*	AF000936	3.1
		
**Apoptosis:**		
*CD27BP*	U82938	6.0
*DR5*	F016268	4.8
		
**Metabolism:**		
*PPAT*	U00238	6.3
*HPRT*	P00492	5.4
		
**Immune response:**		
*MHC class I*	U65416	8.4
**Replication/repair/transcription:**		
*CHAF1A*	U20979	- 12.3
*NEK3*	Z29067	- 5.6
*BTG*	U72649	8.9
*HRC1*	M91083	6.2
*TOP1*	J03250	4.9
*CLK1*	L29222	4.0
		
**Functionally unclassified**		
*PIG7*	AF010312	- 4.9
*menin*	U93236	3.1

*Ratio of normalized data from antibody-treated versus untreated BT474 cells as described in the Materials and Methods section. Negative values indicate downregulated and positive values upregulated genes.

## Discussion

Here, we investigated whether an immunomagnetic enrichment procedure for micrometastatic cancer cells present in BM aspirates leads to significant changes in the gene expression pattern of the enriched tumor cells. In order to mimic the biological conditions of a tumor type with frequent BM involvement, we used two breast cancer cell lines (MCF-7 and BT474). Both cell lines expressed the target antigens, EpCAM and HER2/neu, for immunomagnetic separation at different levels [12] and they were incubated with the anti-EpCAM and anti-HER2/neu antibodies according to the same immunomagnetic enrichment protocol used for the BM samples from cancer patients analyzed recently [9]. It has been shown by other groups that some of the mAb directed against HER2/neu (e.g., Herceptin^R^) can specifically block cell proliferation and affect gene expression in HER2/neu-positive breast cancer cells [13,14]. Furthermore the incubation of human cells with anti-EpCAM-specific mAbs (e.g. KS1/4 mAb) can induce considerable changes in the expression of insulin and glucagons [15]. However, our present results suggest that the two antibodies against EpCAM and HER2/neu used for the immunomagnetic selection process did not considerably influence the gene expression pattern of the enriched cells, although the HER2/neu- positive cell line showed a slightly increased number of differentially expressed genes. These genes are involved in extracellular matrix remodeling, signal transduction and replication, repair and transcription, and they were either up or downregulated after antibody incubation. For example, HER2/neu gene expression was downregulated after antibody binding, as expected from reports in the literature [16]. Taken together, we cannot exclude subtle changes in the expression of individual genes after antibody incubation, but we observed no obvious shift in the expression pattern that exceeds the normal variability of duplicate experiment.

Thus, we conclude that the immunomagnetic selection protocol described here might be useful for experimental approaches aimed to determine the gene expression profile and genome of disseminated CK-positive cells [17,18]. As we performed our study only on two breast cancer cell lines, larger series of similar experiments with further cancer cell lines as well as with enriched tumor cells from the blood or BM must be investigated to draw firm conclusions. The detection and characterization of micrometastatic cancer cells will provide new insights into the biology of the metastatic process in cancer patients. This will lead to an improved molecular staging of cancer patients and to the identification of new biological targets for adjuvant systemic therapies aimed to eradicate micrometastatic disease before the onset of overt metastasis signals incurability.
